# Whole genome analyses of toxicants tolerance genes of *Apis mellifera* gut-derived *Enterococcus faecium* strains

**DOI:** 10.1186/s12864-023-09590-0

**Published:** 2023-08-24

**Authors:** Heba A. H. Zaghloul, Nancy M. El Halfawy

**Affiliations:** Department of Botany and Microbiology, Faculty of Science, Alexandria University, Moharam Bek 21511, Alexandria, Egypt

**Keywords:** Heavy metals, Xenobiotics, Comparative genomics, Honeybee gut, *Enterococcus faecium*, *Apis mellifera*.

## Abstract

**Background:**

Because of its social nature, the honeybee is regularly exposed to environmental toxicants such as heavy metals and xenobiotics. These toxicants are known to exert strong selective pressure on the gut microbiome’s structure and diversity. For example, resistant microbial members are more likely to dominate in maintaining a stable microbiome, which is critical for bee health. Therefore, the aim of this study was to examine the *Enterococcus faecium* strains isolated from bee guts for their in vitro growth and tolerability to diverse heavy metals and xenobiotics. An additional aim was to analyze the genomes of *E. faecium* isolates to assess the molecular bases of resistance and compare them with *E. faecium* species isolated from other environmental sources.

**Results:**

The *E. faecium* bee isolates were able to tolerate high levels (up to 200 mg/L) of toxicants, including cadmium, zinc, benzoate, phenol and hexane. Moreover, the isolates could tolerate toluene and copper at up to 100 mg/L. The genome of *E. faecium* Am5, isolated from the larval stage of *Apis mellifera* gut, was about 2.7 Mb in size, had a GC content of 37.9% and 2,827 predicted coding sequences. Overall, the Am5 genome features were comparable with previously sequenced bee-gut isolates, *E. faecium* Am1, Bee9, SM21, and H7. The genomes of the bee isolates provided insight into the observed heavy metal tolerance. For example, heavy metal tolerance and/or regulation genes were present, including *czcD* (cobalt/zinc/cadmium resistance), *cadA* (exporting ATPase), *cutC* (cytoplasmic copper homeostasis) and *zur* (zinc uptake regulation). Additionally, genes associated with nine KEGG xenobiotic biodegradation pathways were detected, including γ-hexachlorocyclohexane, benzoate, biphenyl, bisphenol A, tetrachloroethene, 1,4-dichlorobenzene, ethylbenzene, trinitrotoluene and caprolactam. Interestingly, a comparative genomics study demonstrated the conservation of toxicant resistance genes across a variety of *E. faecium* counterparts isolated from other environmental sources such as non-human mammals, humans, avians, and marine animals.

**Conclusions:**

Honeybee gut-derived *E. faecium* strains can tolerate a variety of heavy metals. Moreover, their genomes encode many xenobiotic biodegradation pathways. Further research is required to examine *E. faecium* strains potential to boost host resistance to environmental toxins.

**Supplementary Information:**

The online version contains supplementary material available at 10.1186/s12864-023-09590-0.

## Background

Globally, heavy metals and xenobiotic contamination constitute a considerable environmental problem because of their high toxicity and long persistence [1]. Heavy metals have been present in the Earth’s crust since its formation. However, the tremendous increase in heavy metal use has resulted in the accumulation of metallic compounds in the environment. Anthropogenic activity is the primary source of heavy metal contamination, with insecticides and fertilizers playing a secondary role [1, 2]. According to previous studies, environmental pollutants and heavy metals can have devastating effects on bees and other economically important insects, such as the silkworm, *Bombyx mori* [2, 3]. For example, bee exposure to heavy metals can induce cell apoptosis, mutations, neurotoxic effects, and immunodeficiency [4]. Additionally, other environmental toxicants, like xenobiotics, are associated with similar devastating effects. Xenobiotics include pesticides, nitroaromatic compounds, phenolics, halogenated compounds, polycyclic aromatic hydrocarbons (PAHs), chlorinated compounds and other industrial chemicals. These compounds have complex structures that make them challenging to degrade [5]. Once they are released into the environment, xenobiotics can bioaccumulate within the food chain due to their affinity for organic substances, causing toxic adverse effects on natural ecosystems, humans, animals, and economic insects [6]. Furthermore, unintended effects have been reported on the host-associated microbiome [7].

The honeybee (*Apis mellifera* L.) is a social insect that is exposed to many heavy metals and xenobiotics, some of which accumulate (particularly in beehives) when located near industrial areas [8, 9]. The gut microbiome has an important role as it offers metabolic and protective functions for honeybee health [10]. Overall, heavy metals and xenobiotics exert a strong selective pressure on gut microbiome structure and diversity. The loss of key symbiotic bacterial members was found to impair bee immunity and nutrition and increase xenobiotic toxicity [11]. For example, pre-existing resistant bacteria make up a large proportion of the bee population and new resistant strains can be selected. Furthermore, bacterial gene transfer is commonly observed in conjunction with this phenomenon [12].

Indeed, numerous gut-associated lactic acid bacteria (LAB) can remove heavy metals efficiently through biosorption and bioaccumulation mechanisms [13]. *Enterococcus faecium* is a normal LAB inhabitant of the honeybee gastrointestinal tract (GIT) and has the ability to persist in the bee gut in the presence of hazardous contaminants [14]. Despite the large number of *Enterococcus* genomes available in the databases, there is very limited information for *E. faecium* isolated from honeybee guts. Further research is needed to investigate the genome, potential benefits, and environmental applications of such isolates. In the current study, the tolerability of bee-gut-derived *E. faecium* isolates to diverse heavy metals and xenobiotics was examined in vitro. Furthermore, the whole genome of the *E. faecium* Am5 honeybee gut isolate was sequenced. The genome features and phylogenetic relatedness of the Am5 isolate were compared to previously sequenced bee-gut-derived isolates. Furthermore, heavy-metal resistance genes, genes involved in combating xenobiotics and biodegradation pathways were identified. Overall, the results provide evidence that honeybee-gut-derived *E. faecium* strains can tolerate a variety of heavy metals and possess a broad range of xenobiotic biodegradation pathways encoded in their genome.

## Results

### Identification and characterization of *E. faecium* Am5

**Fig. 1:**
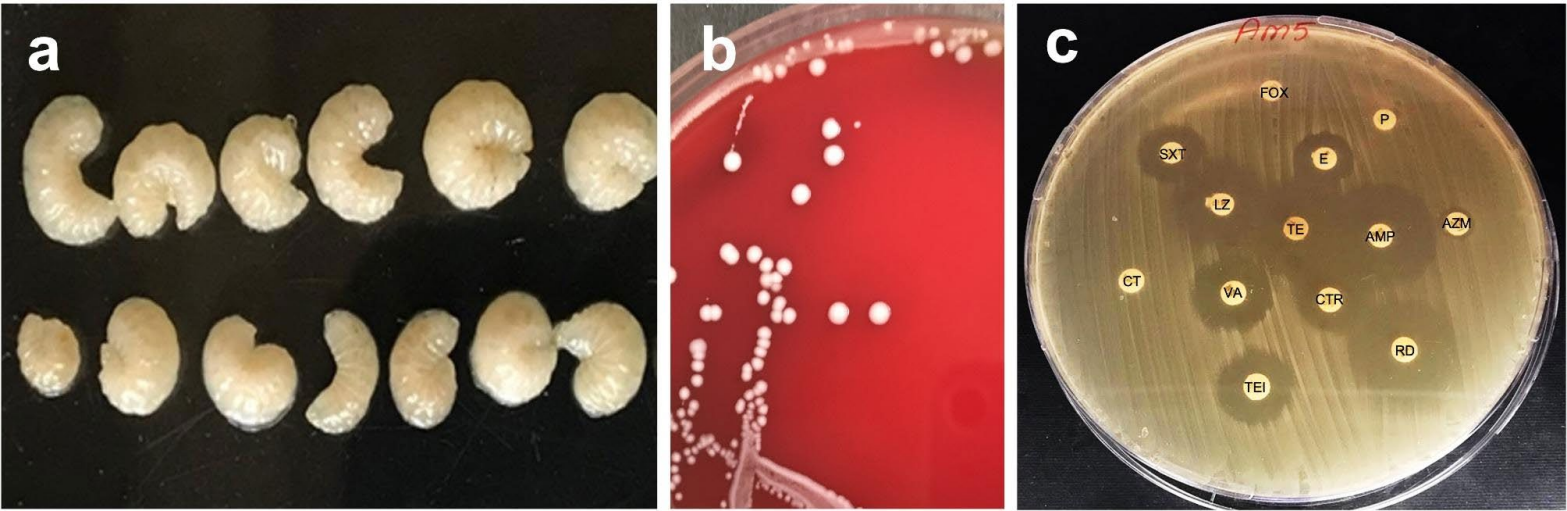
**(a)** *Apis mellifera* larvae; **(b)** colony morphology of *Enterococcus faecium* strain Am5 on the surface of blood agar after 24 h of incubation at 37ºC; **(c)** Antibiotic susceptibility result of Am5

Strain Am5 was isolated from the gut of honeybee larvae (Fig. 1a), and the microscopic examination revealed that the cells are coccoid. Furthermore, biochemical characterization using the VITEK 2 system identified the Am5 isolate as *E. faecium* with a 99% probability. The growth of the Am5 strain on blood agar plates revealed a phenotypic absence of hemolytic activity and it was identified as a gamma-hemolytic bacterium (Fig. 1b).

### Antimicrobial susceptibility testing (AST)

The antibiotic susceptibility revealed that Am5 is susceptible to the following antibiotics: vancomycin, tetracycline, teicoplanin, and ampicillin. Moreover, Am5 showed intermediate sensitivity to linezolid and erythromycin. Am5 was resistant against penicillin G, cefoxitin, azithromycin and colistin sulphate (Fig. 1c). The minimum inhibitory concentrations (MIC) were interpreted using the VITEK 2 system according to CLSI 2017 guidelines and Am5 was found to be susceptible to the following antibiotics (in µg/mL): ampicillin (≤ 2), imipenem (≤ 1), ciprofloxacin (≤ 0.5), teicoplanin (≤ 0.5), vancomycin (≤ 0.5), tetracycline (≤ 1), and tigecycline (≤ 0.12). Furthermore, Am5 revealed intermediate sensitivity to erythromycin and linezolid.

### General features of the Am5 draft genome

**Fig. 2:**
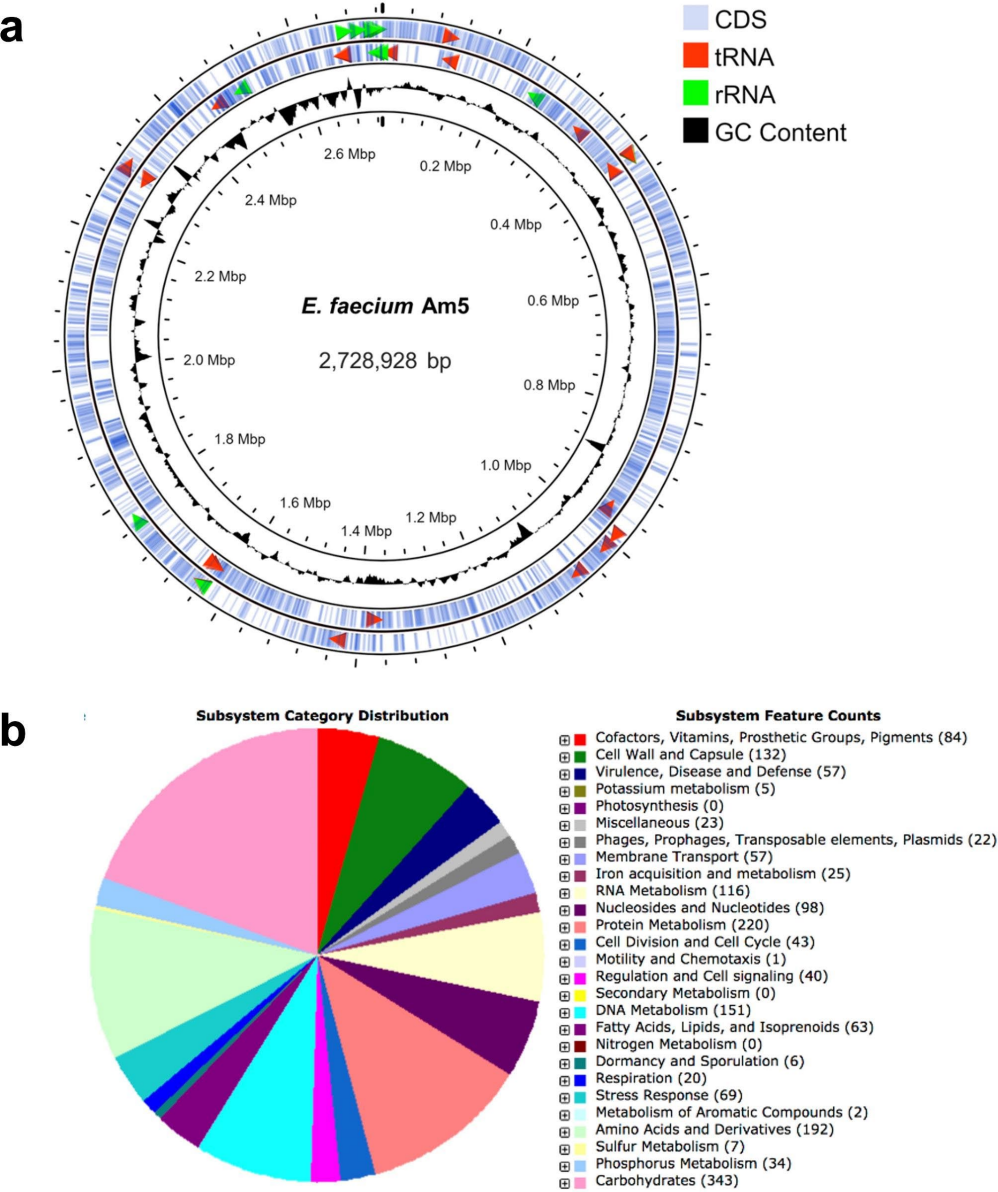
**(a)** Circular draft genomic map of the *Enterococcus faecium* strain Am5 contig sequences. The genome is 2,728,928 bp in size and has a G + C content of 37.9%. The tRNA genes are denoted as red arrows, and the rRNA genes are denoted as green arrows. Image was generated using Proksee (https://proksee.ca/); **(b)** subsystem categories and feature distribution of the *E. faecium* Am5 genome based on the RASTk annotation server

Whole-genome sequencing (WGS) of *E. faecium* Am5 was obtained using the Illumina Hi-Seq platform (Illumina, USA) with 30X sequence coverage, yielding 453,383 reads with a median insert size of 675 bases and contigs with an N50 value of 87,955 bp. The reads were *de novo* assembled, yielding 83 large contigs (> 1,000 bp). The general genomic features indicated a total genome length of 2.7 Mb (Fig. 2a), with an estimated 2,827 CDS regions and a G + C content of 37.9%. A total of 63 tRNA genes and 6 rRNA genes were predicted using the BV-BRC server. The draft genome sequence of the Am5 isolate was deposited at DDBJ/ENA/GenBank under the accession number JAHLTK010000000.

The distribution of Clusters of Orthologous Groups (COG) categories in the *E. faecium* Am5 genome was predicted using the Rapid Annotations using Subsystems Technology (RASTk) annotation server (Fig. 2b). The analysis revealed that functional categories like carbohydrates, protein metabolism and amino acid-related genes were the most abundant in the Am5 genome.

### Phylogenetic relationships and genomic correlation analysis

**Fig. 3:**
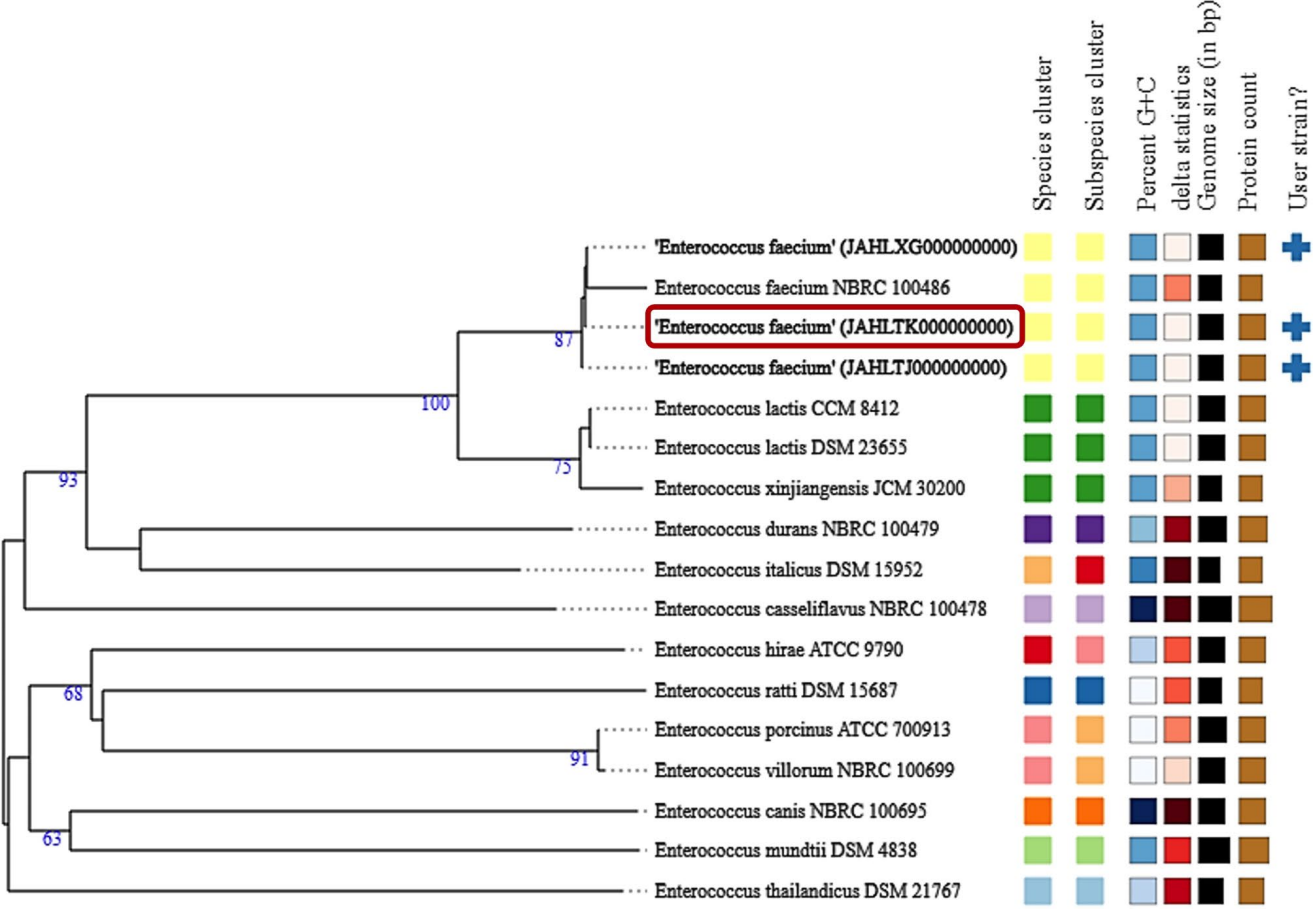
Phylogenomic tree of *Enterococcus faecium* Am1 (JAHLTJ000000000), Am5 (JAHLTK000000000), Bee9 (JAHLXG000000000) and 14 other *Enterococcus* spp. Branch lengths were scaled in terms of GBDP distances formula *d5*. FastME 2.1.6.1 was used to infer a tree from GBDP distance calculated from genome sequences. The numbers above the branches are GBDP pseudo-bootstrap support values > 60% from 100 replications, with an average branch support of 62.1%. The tree was rooted at the midpoint. Leaf labels with different colours indicate species and subspecies clusters. The tree was constructed with the TYGS webserver (https://tygs.dsmz.de/)

A whole genome-based taxonomic analysis was performed to obtain insights into the phylogenetic relationship between bee gut isolates (Am1, Am5, and Bee9) and other *E. faecium* strains. The Genome BLAST Distance Phylogeny (GBDP) approach was used, and the results revealed that *E. faecium* isolates Am1, Am5 and Bee9 are closely related and cluster together with *E. faecium* NRBC 100,486. As shown in Fig. 3, most features between the three bee gut isolates were largely identical.

### Pathogenicity analysis

Pathogenicity analysis indicated that *E. faecium* Am5 is a non-human pathogen. ARG analysis identified genes associated with low-level resistance (*acc*(6’)-Ii, *liaS*, *liaR*, *efrB*, *adeC*, *efmA*, *msrC*, *dfrE*), but no clinically relevant antibiotic resistance genes (such as vancomycin resistance) were identified. Virulence factor analysis revealed six genes associated with adherence (*ebpA*, *ebpB*, *ebpC*, *strC*, *efaA*, and *scm*), three involved in antiphagocytosis (*cpsA/uppS*, *cpsB/cdsA*, and *cpsC*), one biofilm formation gene (*bopD*), and an immune evasion-associated factor (*eps3*). Importantly, the genome was void of the critical virulence genes associated with pathogenicity, such as *gelE* (gelatinase), *esp* (gene encoding surface protein), *agg* (aggregation) and *ace* (adhesion collagen protein). Additionally, no toxin-related genes were predicted in the genome, such as cytolysin genes (*cylA*, *cylB*, *cylL*, *cylM*, *cylR1*, *cylR2*).

Two plasmids were predicted from the Am5 genome data, namely rep29 (accession number HQ696461) and repUS15 (accession number CP004064). These plasmid sequences were free of critical virulence and antibiotic-resistance genes present in pathogenic *E. faecium* strains. Moreover, the PHASTER tool identified one intact (PHAGE_Entero_IME_EFm5, 16.7 kb) and three incomplete (PHAGE_Lister_A500, 10.1 kb; PHAGE_Entero_phiFL3A, 15.3 kb; and PHAGE_Escher_RCS47, 15.1 kb) prophage genomes for Am5. Insertion sequence (IS) elements were predicted with ISfinder and most were found to belong to the IS*3* and IS*6* families. The isolates showed the absence of IS*16*, ISEfa11, and ISEfa5 elements, which are associated with pathogenic *E. faecium* strains.

### Prediction of xenobiotic biodegradation genes

**Fig. 4:**
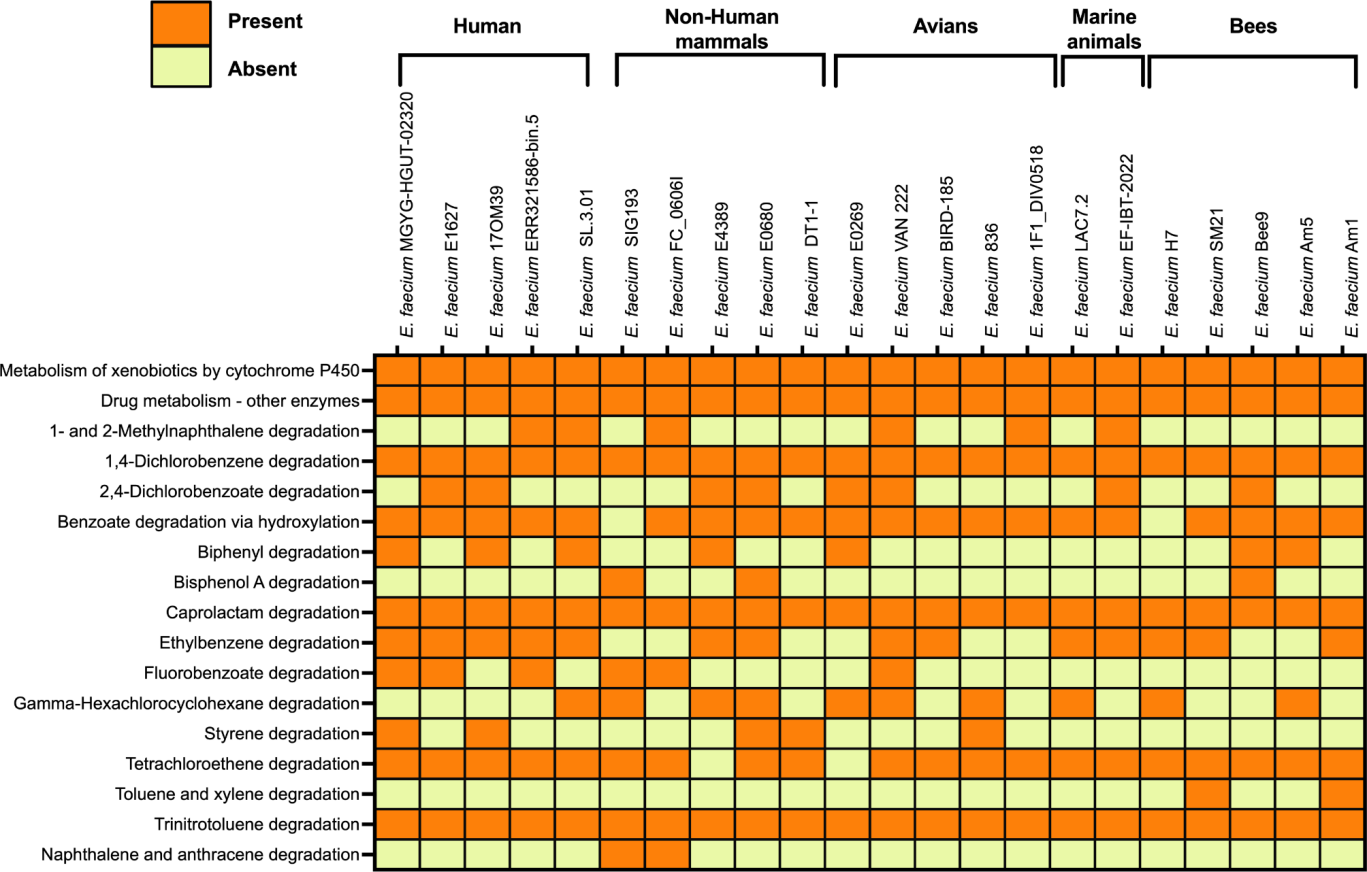
Heatmap of xenobiotic degradation pathways predicted with BV-BRC annotation for isolated bee gut *Enterococcus faecium* strains and other *Enterococcus* sp. from different environmental sources included in the analysis

Numerous gene ontology (GO) pathways associated with xenobiotic biodegradation in bee gut-derived *E. faecium* genomes were predicted using the Kyoto Encyclopedia of Genes and Genomes (KEGG) database. To compare the overall xenobiotic metabolic potential of *E. faecium* Am1, Am5, and Bee9 strains with that of other 19 selected *Enterococcus* strains from various environmental sources, a matrix of predicted pathways was generated and graphically summarized as a heat map (Fig. 4). Several genes involved in significant groups of xenobiotic biodegradation pathways, such as naphthalene, anthracene, and 1- and 2-methylnaphthalene, were found in Am5 genome (Supplementary Table S1). Genetic evidence for the degradation of aromatic and chlorinated aromatic compounds, including toluene, xylene, trinitrotoluene, 1,4-dichlorobenzene, and 2,4-dichlorobenzoate, was detected in the bee gut-derived *E. faecium* genomes (Supplementary Table S2).

### Prediction of heavy metal tolerance and/or regulation genes

**Fig. 5:**
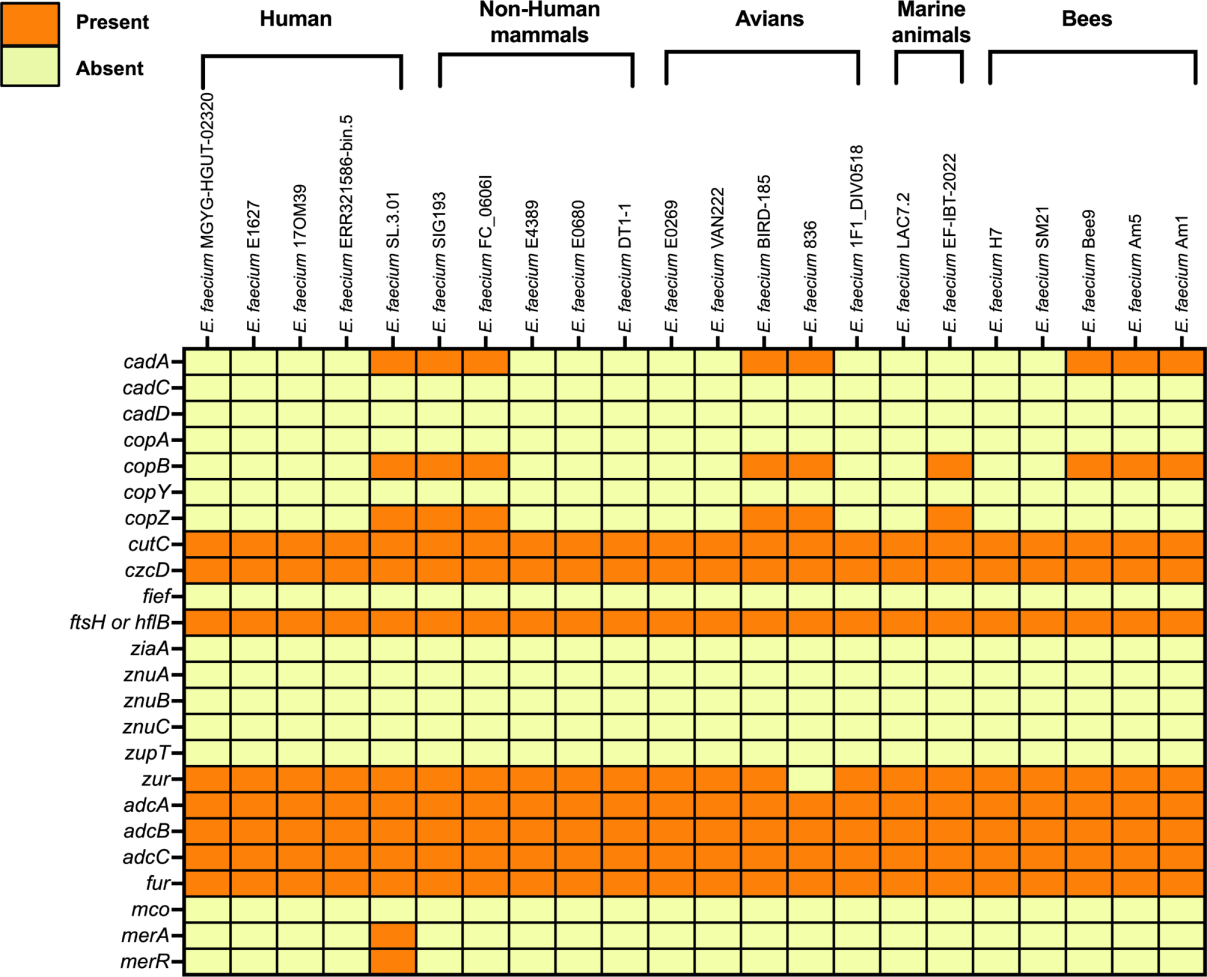
Heatmap of heavy metal tolerance and/or regulation genes predicted with BV-BRC annotation for bee gut isolated *Enterococcus faecium* strains and other *Enterococcus* sp. from different environmental sources included in the analysis

The draft genomes of Am1, Am5 and Bee9 revealed the presence of multiple heavy-metal tolerance genes. Such genes were predicted *in silico* from the genomic data with the BV-BRC webserver and BLASTp. The *cadA* and *copB* genes were predicted in the Am1, Am5, and Bee9 genomes; these genes are involved in cadmium and copper transport, respectively. Moreover, the *czcD* gene was also identified; this gene is associated with cobalt/zinc/cadmium resistance. Furthermore, the *zur* and *fur* genes involved in zinc and iron regulator control, respectively, were also predicted. A set of genes (*adcA*, *adcB*, and *adcC*) associated with zinc transport and *ftsH*, an ATP-dependent zinc metalloprotease, was predicted. Interestingly, the comparative genomic analysis revealed that all the included *E. faecium* strains, regardless of the isolation source, possessed a set of eight heavy-metal tolerance and/or regulation genes, namely, *cutC, czcD, ftsH/hflB, zur, adcA, adcB, adcC* and *fur*. Only the *zur* gene was absent in a single *E. faecium* strain 836 isolated from poultry feces (Fig. 5).

### Tolerance of bee isolates to different concentrations of selected xenobiotics and heavy metals

Heavy metals and xenobiotic tolerance of the three bee-gut derived *E. faecium* strains were assessed by growing them on supplemented MRS broth (Table 1). The results revealed that Am1, Am5 and Bee9 isolates were tolerant to benzoate, phenol, hexane, cadmium and zinc at concentrations up to 200 mg/L. Furthermore, the isolates tolerated toluene and copper up to 100 mg/L. Moreover, Am1 and Am5 isolates were tolerant to benzene up to 100 mg/L. However, isolate Bee9 exhibited greater tolerability upon exposure to 200 mg/L of benzene. Complete growth inhibition was observed when the Am1 isolate was exposed to 50 mg/L of xylene. On the contrary, Am5 and Bee9 isolates were tolerant to xylene up to 200 mg/L.

**Table 1:** Growth of different bee gut *E. faecium* isolates (Am1, Am5 and Bee9) on different concentrations (mg/L) of xenobiotics and heavy metals

	Concentration of toxicant (mg/L)								
	*E. faecium* Am1			*E. faecium* Am5			*E. faecium* Bee9		
	50	100	200	50	100	200	50	100	200
Benzene	**+**	**+**	**-**	**+**	**+**	**-**	**+**	**+**	**+**
Xylene	**-**	**-**	**-**	**+**	**+**	**+**	**+**	**+**	**+**
Toluene	**+**	**+**	**-**	**+**	**+**	**-**	**+**	**+**	**-**
Benzoate	**+**	**+**	**+**	**+**	**+**	**+**	**+**	**+**	**+**
Phenol	**+**	**+**	**+**	**+**	**+**	**+**	**+**	**+**	**+**
Hexane	**+**	**+**	**+**	**+**	**+**	**+**	**+**	**+**	**+**
Cadmium	**+**	**+**	**+**	**+**	**+**	**+**	**+**	**+**	**+**
Zinc	**+**	**+**	**+**	**+**	**+**	**+**	**+**	**+**	**+**
Copper	**+**	**+**	**-**	**+**	**+**	**-**	**+**	**+**	**-**

Growth (+), no growth (-)

### Distribution of Zn, Cu, and Cd metals in *E. faecium* Am5

Transmission electron microscopy (TEM) was performed to investigate the capability of *E. faecium* Am5 to bioaccumulate or bind with the heavy metals under investigation. TEM micrographs revealed that the extracellular surface of untreated (metals-free) bacterial cells was intact, and the cells had a homogenous cytoplasm (Supplementary Fig. 1a). Moreover, changes in cell surface morphology and visible deposits adsorbed on the extracellular surfaces of bacterial cells were observed with Zn-, Cu-, and Cd-treated cells after 24 h of incubation (Supplementary Fig. 1b, c & d). Additionally, visible dark areas of metal deposits occupied many areas inside the cell cytoplasm of Cu- and Cd-treated cells (Supplementary Fig. 1c & d).

### EDX analysis

**Fig. 6:**
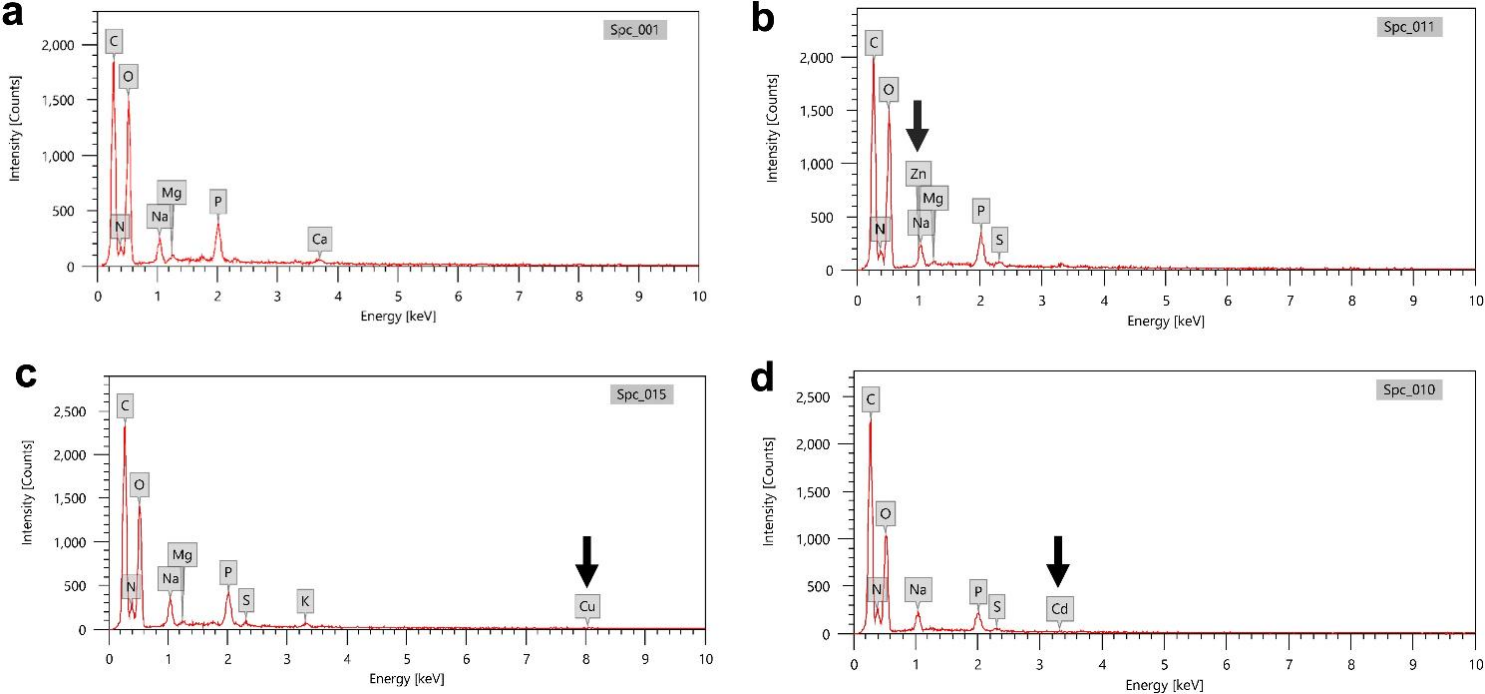
Energy Dispersive X-ray Spectroscopy (EDX) analysis of *Enterococcus faecium* Am5 with the following treatments: **(a)** control-untreated cells; **(b)** Zn-treated cells; **(c)** Cu-treated cells; and **(d)** Cd-treated cells

Energy dispersive X-ray spectroscopy (EDX) was used to confirm the biosorption of the heavy metals on the bacterial cell surfaces. No heavy metal-related peaks were detected in the untreated sample (Fig. 6a). Meanwhile, the qualitative elemental analysis revealed the presence of Zn, Cu and Cd peaks in Zn-, Cu-, and Cd-treated cells with mass ratios of 0.31, 0.64 and 0.16% (w/w), respectively (Fig. 6b, c & d).

## Discussion

When foraging in the field or feeding on a contaminated meal, bees are exposed to a variety of toxicants, including heavy metals and xenobiotics. A sublethal dose of these toxicants was found to alter the behavior and growth of both individual and colony honeybees. It was proposed that the ability of bees to defend themselves against these toxins is mediated by gut-associated microbiota or bee enzymes such as cytochrome P450 [15]. Recently, a growing number of studies have demonstrated the close link between gut microbiota and bee health. For example, the bee’s gut bacteria were found to be associated with host nutrition, weight gain, endocrine signaling, stimulating the immune system and protecting the host from pathogen colonization [16–18]. On the other hand, research on the bee gut microbiota’s function in heavy metal resistance or xenobiotic detoxification is scarce.

In the current study, the growth of *E. faecium* Am5 isolated from *Apis mellifera* larvae was assessed in the presence of heavy metals (cadmium, zinc, and copper) and xenobiotics (benzene, toluene, xylene, benzoate, phenol, and hexane). Moreover, two bee-gut associated isolates (Am1 and Bee9), identified as *E. faecium* species using biochemical and molecular approaches [19], were also examined. Interestingly, the three isolates (Am5, Am1 and Bee9) were able to survive in the presence of most of these toxicants. Extremely high toxicant concentrations were avoided because, as recommended [20], it is preferable to use environmentally relevant concentrations that to which the host organism is known to be exposed rather than adding excessively high concentrations that are not present in vivo. Only xylene was found to be toxic to the Am1 isolate, despite the presence of metabolic pathways associated with toluene and xylene degradation. Indeed, the presence of genes associated with the degradation pathway is not evidence for catabolic function. Moreover, it was reported that the toxicity of xylene could be higher than that of benzene because of the two methyl substitutions in the benzene nucleus [21].

WGS was employed to identify the genetic background associated with the ability of the *E. faecium* Am5 strain to resist heavy metals or detoxify xenobiotics. The previously sequenced bee-gut isolates, *E. faecium* Am1 (NCBI accession no. JAHLTJ000000000) and *E. faecium* Bee9 (NCBI accession no. JAHLXG000000000) [19], *E. faecium* SM21 (NCBI accession no. NZSDXT00000000) [14] and *E. faecium* H7 (NCBI accession no. CP083179) [22], were also included in this analysis. Furthermore, a set of *E. faecium* strains derived from the guts of other environmental sources, such as humans, non-human mammals, avians and marine animals, were included in this comparative genomics study. The comparison between Am1, Am5 and Bee9 revealed similarities between these strains on a molecular and biological function level. Interestingly, the three bee-gut strains possess many genes for the metabolism of carbohydrate- and protein-rich meals (Fig. 2). This dominance of carbohydrate and protein metabolism genes emphasizes the critical role *E. faecium* plays in aiding the host’s nutrition in nature. Furthermore, the three strains share the same virulence and antibiotic resistance gene profiles. This result was surprising given that Am1 and Am5 strains were isolated from *Apis mellifera* larval stages and Bee9 was isolated from the adult stage [19]. The presence of the same virulence and antibiotic resistance profiles in the three gut-inhabitant *E. faecium* strains, regardless of the insect’s growth stage, may reflect the importance of these genes for this bacterial species to survive in this unique habitat.

On the other hand, the analysis of the heavy-metal resistome in bee isolates resulted in the identification of a set of genes, including *cadA*, *copB*, and *czcD*, which are responsible for cadmium, copper and zinc resistance, respectively, at the molecular level. CadA (also reported in *E. faecium* UC7251 [23]) encodes a Cd^2+^/ATPase protein transporter, which aids in heavy-metal stress adaptation. CopB is a P-type ATPase responsible for ATP-dependent Cu transport across the cytoplasmic membrane [24, 25]. The *czcD* gene is linked to cobalt, zinc and cadmium resistance [25]. Despite the importance of zinc as a micronutrient, exposure to an excessive amount can cause toxicity in cells and disruption of essential biological functions [26]. Additionally, the *E. faecium* Am5 isolate was able to accumulate toxic metals within large particles (Supplementary Fig. 1). Indeed, the exopolysaccharide-cell surface binding capabilities of *E. faecium* Am5 could be the reason why these hazardous metals were accumulated. Generally, the most commonly reported mechanisms adopted by bacteria, including LAB, to remove heavy metals are biosorption and bioaccumulation. Heavy metals can bind to cell walls by biosorption, whereas they can pass through the cell wall and accumulate inside cells through bioaccumulation [27–29].

Similarly, the xenobiotic detoxification genes analysis indicated the presence of nine xenobiotic biodegradation pathways. Insects detoxify xenobiotics in their guts through a number of processes. Before excretion, they use a combination of enzymes (monooxygenases and esterases) to cleave or modify the xenobiotic. Previous research found that *Enterococcus* (Firmicutes) gut bacteria are responsible for *Spodoptera frugiperda* resistance to a variety of pesticides, including chlorpyrifos and deltamethrin [30]. Similarly, several insects gut symbiotic bacterial species assisted their hosts’ ability to detoxify pesticides such as benzoylurea, methoprene, neonicotinoid, carbamate, and organochloride [31]. The microbial enzymes in their guts, on the other hand, were found to contribute to the breakdown process. The bacteria were able to use the hydrolysis products for their growth. In general, xenobiotic identification and uptake occur via passive diffusion and active transport, both of which are part of bacteria’s molecular stress response mechanism [32]. When xenobiotics enter cells, catabolic genes are activated and detoxified by enzymes such as phenolic acid decarboxylase (PadC), nitrilotriacetate monooxygenase, alcohol dehydrogenase, and carboxylesterase [33, 34]. This combination of eukaryotic and prokaryotic enzymatic activity is critical for xenobiotic metabolization success [31, 35]. On the other hand, the biotransformation of the parent xenobiotic into other forms by these enzymatic activities is not always environmentally safe. A recent study reported the microbial biotransformation of the insecticide chlorpyrifos into the more toxic chlorpyrifos oxon form by the gut microbiota of *Drosophila melanogaster*. Chlorpyrifos oxon has a 10- to 100-fold greater inhibition of acetylcholinesterase and hence is higher in neurotoxicity. Therefore, the biological and environmental consequences of pesticides on off-target species should take into consideration the microbiota composition of these species and their impact [36].

The presence of several heavy-metal and xenobiotic resistance genes in the *E. faecium* species isolated from various environmental sources (Figs. 4 and 5) was unexpected. This may, however, indicate the vital role that this bacterial species plays in protecting its host from an increasing number of toxicants. There are some claims that microflora evolve faster than host insects, resulting in rapid insect adaptation to pesticides through mutualistic microorganisms [31]. More in-depth research is needed to determine the role of these *E. faecium* anti-toxicant genes in the relevant host and their indirect environmental impacts.

## Conclusions

Our results demonstrate that *E. faecium* honeybee gut isolates can tolerate a variety of heavy metals. Furthermore, key genes for heavy-metal tolerance and xenobiotic degradation pathways were predicted in the genomes of *E. faecium* species. Future in vivo studies are needed to ascertain whether *E. faecium* can improve host tolerance to heavy metals and xenobiotics. Understanding the fate of pollutants in our environment enables us to develop more effective future protection strategies.

## Methods

### Bacterial strain and culture condition

*E. faecium* Am5 was isolated from *Apis mellifera* larvae gut provided through the Faculty of Agriculture, University of Alexandria, Egypt. The triturated gut was cultivated on De Man-Rogosa-Sharpe (MRS; HiMedia, India) at 37 °C for 24 h. The Am5 isolate was biochemically identified using a VITEK 2 GP ID card (BioMérieux, France). Pure bacterial culture was stored at -20 °C in MRS broth supplemented with 50% (v/v) glycerol until further use. To investigate hemolytic activity, Am5 was cultivated on 5% (v/v) blood agar plates and incubated at 37 °C for 24 h.

### Antimicrobial susceptibility testing (AST)

The antibiotic sensitivity test was carried out on Muller Hinton agar (MHA; HiMedia, India) plates containing antibiotic discs in triplicate. The plates were incubated at 37 °C for 24 h, and the results were expressed in millimeters (mm) of inhibition. AST was performed using the GP susceptibility card AST-P592 (BioMérieux; https://www.biomerieux.com) and the VITEK 2 system (version 9.02), according to the manufacturer´s guidelines. The MIC values of strain Am5 were classified as susceptible (S), intermediate (I), or resistant (R) according to the CLSI recommendations.

### Whole-genome sequencing and assembly

Genomic DNA was isolated using the GeneJET Genomic DNA Purification Kit (Thermo Fisher Scientific, UK), following the manufacturer’s instructions for Gram-positive bacterial DNA isolation. Whole genome sequencing (WGS) of *E. faecium* Am5 was performed by MicrobesNG (Birmingham, UK; http://microbesng.uk) using the Illumina Hi-Seq platform (Illumina, USA) with paired-end reads of 250 bp in length. Illumina reads were prepared using the Kapa Biosystems Library Quantification Kit for Illumina. Raw sequences were trimmed using Trimmomatic (version 0.30) with a sliding cut-off of Q15 [37]. The SPAdes assembler software (version 3.7.0) [38] was used to reconstruct the genome, and the quality of the genome assemblies was assessed using the Quality Assessment Tool for Genome Assemblies (QUAST) [39].

### Genome annotation and subsystem analysis

Genome annotation was performed using Prokka software (version 1.11) [40], the BV-BRC server (version 3.25.0; https://www.bv-brc.org/) [41], and the NCBI Prokaryotic Genome Annotation Pipeline (PGAP) [42]. Subsystem categories and feature distribution of the *E. faecium* Am5 genome was performed using Rapid Annotations using Subsystems Technology (Annotation scheme: RASTtk) [43].

### Public data acquisition

The genome of the honeybee gut derived Am5 strain was compared with 21 selected *E. faecium* strains. Strains were classified according to their host into Group 1 from insects’ gut, Group 2 from non-human mammals’ feces, Group 3 from human gut, Group 4 from avian feces, and Group 5 from other sources. Supplementary Table 2 lists the NCBI reference sequence accession numbers, host, and geographic location of selected genomes.

### Phylogenomic analysis

For whole genome-based taxonomic analysis, the Type (Strain) Genome Server (TYGS) bioinformatics platform, available through https://tygs.dsmz.de/, was used [44]. Bee isolate Am1, Am5, and Bee9 genomes were compared against type strain genomes available in the TYGS database using the MASH algorithm [45]. Genome BLAST Distance Phylogeny (GBDP) under the algorithm “coverage” and distance formula *d5* [46] was used to calculate precise distances between genomes. The resulting intergenomic distance was used to infer a balanced minimum evolution tree with branch support using FASTME 2.1.6.1 [47]. Branch support was inferred from 100 pseudobootstrap replicates each. The tree was rooted at the midpoint and visualized with PhyD3 [48].

### Pathogenicity analysis of the *E. faecium* Am5 genome

The pathogenicity of *E. faecium* Am5 WGS was predicted using the PathogenFinder online tool provided by the Center for Genomic Epidemiology (https://cge.cbs.dtu.dk) [49]. Antibiotic resistance genes (ARG) were predicted using the Comprehensive Antibiotic Resistance Database (CARD) [50], available through the BV-BRC server (version 3.30.5*i*). Virulence factors in the genome were investigated using the VFanalyzer platform (https://www.mgc.ac.cn), available through the Virulence Factor Database (VFDB). Plasmids and prophage sequences were predicted using PlasmidFinder (version 2.0) and the PHAge Search Tool Enhanced Release web server (PHASTER; https://phaster.ca) [51], respectively. Insertion sequences (IS) and transposons were predicted with the ISfinder server (https://www-is.biotoul.fr) using BLASTn (version 2.2.31+) [52].

### Prediction of xenobiotic biodegradation and heavy metal-related genes

To identify KEGG pathways [53] associated with xenobiotic metabolic pathways and heavy-metal tolerance genes in the Am1, Am5 and Bee9 genomes, the BV-BRC server was used. For specific homology, all translated CDSs previously predicted in genomes were subjected to BLASTp against the GenBank database. To obtain a comparative visualization of the prediction of xenobiotic biodegradation and heavy metal tolerance genes, heat maps were generated using the GraphPad Prism 9 program (https://www.graphpad.com).

### Growth of bee gut isolates at different concentrations of selected xenobiotics and heavy metals

Different selected xenobiotics (benzene, xylene, toluene, benzoate, phenol, and hexane) and heavy metals (cadmium, zinc, and copper) stock solutions with a final concentration of 10 mg/mL were prepared and sterilized using a 0.22 μm bacterial filter. The investigation of xenobiotic biodegradation and heavy metal tolerance was carried out in MRS (Merck, Germany). Test tubes containing 15 mL of MRS broth supplemented with different concentrations of each pollutant (50, 100, and 200 mg/L) were inoculated with a 1% (v/v) overnight culture of bee isolate and closed tightly. Tubes containing the same pollutant concentration served as a negative control, while MRS broth inoculated with an overnight culture of bee gut isolate served as a positive control without pollutant addition. The cultures were incubated statically at 30 °C for 7 days. The bacterial growth was measured with a spectrophotometer at OD_600_ nm, and the results were recorded as (+) for growth and (-) for no growth. The growth was then checked on MRS agar plates to confirm cell viability after treatment.

### Transmission electron microscopy (TEM)

In order to investigate the possible localization of accumulated metals within *E. faecium* strain Am5, the isolate was cultivated on MRS liquid medium supplemented with 200 mg/L of Cd, Zn, and 100 mg/L of Cu. The cell pellets of both untreated (control) and metal-treated cells were collected by centrifugation, washed twice, and fixed by immersing them in phosphate buffer solution (pH 7.2) at 4 °C for 3 h. The samples were then fixed in 2% OsO_4_ at 4 °C for 2 h, washed, and dehydrated using acetone. Samples were then embedded in resin to polymerize, cut into sections, and stained using uranyl acetate for 5 min. Cells were examined with transmission electron microscopy (TEM; JSM-1400 PLUS, JEOL) at the EM Unit, Alexandria University, Egypt.

### Energy dispersive X-ray spectroscopy (EDX)

To validate the biosorption of heavy metals in both treated and untreated cells, energy dispersive X-ray spectroscopy (EDX) at an accelerating voltage of 20 kV was performed. The analysis was performed using scanning electron microscopy (SEM; JSM-IT 200, JEOL) at the EM Unit, Alexandria University, Egypt.

### Electronic supplementary material

Below is the link to the electronic supplementary material.

Supplementary Material 1

Supplementary Material 2

## Data Availability

The whole genome sequence generated in the current study is available in the DDBJ/ENA/GenBank repository, JAHLTK010000000.

## Abbreviations

*E. faecium*: *Enterococcus faecium*
EDX: Energy Dispersive X-ray Spectroscopy
CDS: Coding Sequence
COG: Clusters of Orthologous Groups
GBDP: Genome BLAST Distance Phylogeny
GIT: Gastrointestinal Tract
GO: Gene Ontology
KEGG: Kyoto Encyclopaedia of Genes and Genomes
MRS: De Man–Rogosa–Sharpe
PAH: Polycyclic aromatic hydrocarbons
WGS: Whole Genome Sequencing
