# Supplementary material for: Whole genome analyses of toxicants tolerance genes of Apis mellifera gut-derived Enterococcus faecium strains
Source: BMC Genomics. 2023 Aug 24;24:479. doi: 10.1186/s12864-023-09590-0 (PMC10463970; doi:10.1186/s12864-023-09590-0)
Supplement: Supplementary file 1 — Supplementary Material 1 [file 12864_2023_9590_MOESM1_ESM.docx]

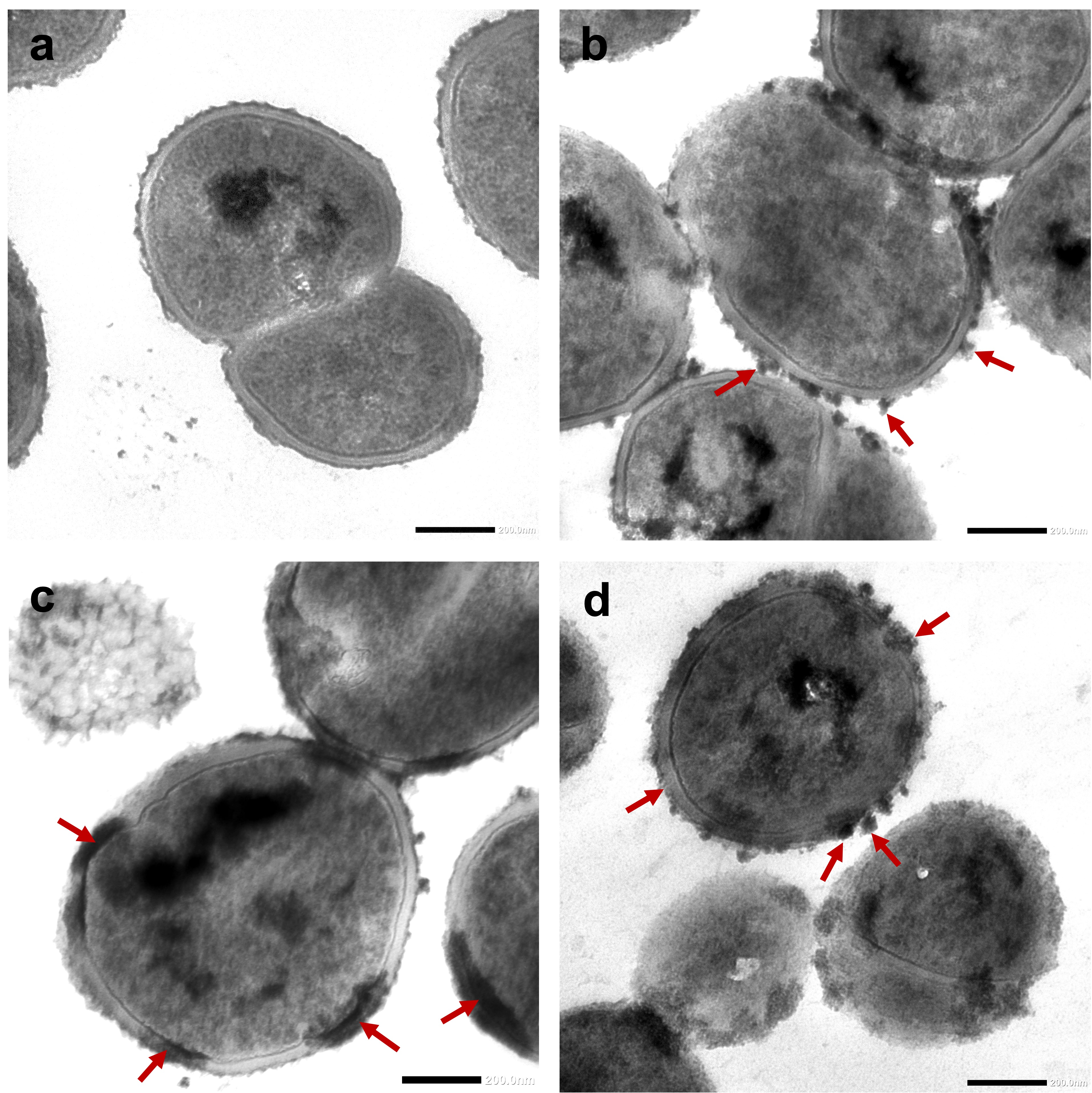


**Supplementary figure 1.** Transmission electron microscopy (TEM) of *Enterococcus faecium* Am5 grown in MRS broth medium with the following treatments **(a)** Control-untreated cells with metals; **(b)** Zn-treated cells; **(c)** Cu-treated cells; and **(d)** Cd-treated cells. The arrows indicate metal accumulation on the bacterial cell wall. Scale bar = 200 nm.
