# Supplementary material for: Whole genome analyses of toxicants tolerance genes of Apis mellifera gut-derived Enterococcus faecium strains
Source: BMC Genomics. 2023 Aug 24;24:479. doi: 10.1186/s12864-023-09590-0 (PMC10463970; doi:10.1186/s12864-023-09590-0)
Supplement: Supplementary file 2 — Supplementary Material 2 [file 12864_2023_9590_MOESM2_ESM.docx]

**Supplementary Table 1.** Metabolic pathways and genes encoding proteins involved in xenobiotics biodegradation in *E. faecium* Am5 genome predicted using BV-BRC server.

| **Pathway name** | **Product** | **EC description** |
| --- | --- | --- |
| γ-Hexachlorocyclohexane degradation | 2-haloalkanoic acid dehalogenase | (S)-2-haloacid dehalogenase |
| Benzoate degradation via hydroxylation | 4-carboxymuconolactone decarboxylase | 4-carboxymuconolactone decarboxylase |
| Biphenyl degradation | Phenolic acid decarboxylase PadC | Carboxylyases |
| Tetrachloroethene degradation | Nitrilotriacetate monooxygenase component B | With NADH or NADPH as one donor, and incorporation of one atom of oxygen |
|  | Bifunctional protein: zinc-containing alcohol dehydrogenase; quinone oxidoreductase (NADPH: quinone reductase) | With NAD(+) or NADP(+) as acceptor |
| 1,4-Dichlorobenzene degradation | Methyltransferase | Methyltransferases |
|  | Acylphosphate phosphohydrolase | Acylphosphatase |
|  | Ribosomal-protein-S18p-alanine acetyltransferase | Transferring groups other than amino-acyl groups |
| Trinitrotoluene degradation | Putative oxidoreductase YcjS, NADH-binding | Oxidoreductases |
|  | Malolactic enzyme | Oxidoreductases |
| Caprolactam degradation | Peptidoglycan N-acetylglucosamine deacetylase | In linear amides |
|  | Aromatic amino acid aminotransferase gamma @ N-acetyl-L,L-diaminopimelate aminotransferase | Transaminases (aminotransferases) |
| Metabolism of xenobiotics by cytochrome P450 | Alcohol dehydrogenase | Alcohol dehydrogenase |
| Drug metabolism - other enzymes | Carboxylesterase | Carboxylesterase |
|  | Orotate phosphoribosyltransferase | Orotate phosphoribosyltransferase |
|  | Thymidine kinase | Thymidine kinase |
|  | Cytidine deaminase | Cytidine deaminase |
|  | Uridine kinase | Uridine kinase |
|  | Hypoxanthine-guanine phosphoribosyltransferase | Hypoxanthine phosphoribosyltransferase |
|  | Inosine-5'-monophosphate dehydrogenase / CBS domain | IMP dehydrogenase |
|  | GMP synthase [glutamine-hydrolyzing], amidotransferase subunit | GMP synthase (glutamine-hydrolyzing) |

**Supplementary Table 2.** Bacterial strains used in this study including GenBank accession number.

| **Host** | **Strain** | **Source** | **Geographic location** | **Accession number*** |
| --- | --- | --- | --- | --- |
| **Insects** | Am5 | Bee gut Larvae | Egypt | [JAHLTK000000000](https://www.ncbi.nlm.nih.gov/nuccore/JAHLTK000000000) |
|  | Am1 | Bee gut Larvae | Egypt | JAHLTJ000000000 |
|  | Bee9 | Bee gut Adult | Egypt | JAHLXG000000000 |
|  | SM21 | Bee gut | Argentina | [NZSDXT00000000](http://www.ncbi.nlm.nih.gov/biosample/SAMN10845504) |
|  | H7 | Bee gut | China | CP083179 |
| **Non-Human Mammals** | E0680 | Pig faeces | Germany | [AHWN00000000](http://www.ncbi.nlm.nih.gov/nuccore/AHWN00000000) |
|  | E4389 | Dog faeces | Denmark | AHYJ00000000 |
|  | FC_0606I | Cow faeces | Canada | JABTDE000000000 |
|  | SIG193 | Goat faeces | USA | SVAD00000000 |
|  | DT1-1 | Cattle faeces | China | CP050255 |
| **Human** | E1627 | Human gut | Netherlands | AHXJ00000000 |
|  | MGYG-HGUT-02320 | Human gut | Ireland | CABMGT000000000 |
|  | SL.3.01 | Human gut | USA | JAJDLD000000000 |
|  | ERR321586-bin.5 | Human gut | Denmark | CAJLLM000000000 |
|  | 17OM39 | Human gut | India | LWHF00000000 |
| **Avian** | 836 | Poultry faeces | Spain | DYZG00000000 |
|  | BIRD-185 | Dove faeces | Estonia | JACZCA0100000 |
|  | E0269 | Turkey faeces | Netherlands | AHWK00000000 |
|  | VAN 222 | Chicken faeces | Denmark | AIUX00000000 |
|  | 1F1_DIV0518 | Sparrow faeces | Germany | NGLS00000000 |
| **Others** | LAC7.2 | Tilapia gut | Brazil | CP045012 |
|  | EF-IBT-2022 | Pacific White shrimp gut | Mexico | CP101669 |

*****All accession numbers are from GenBank.
